# Dietary Melanoidins from Biscuits and Bread Crust Alter the Structure and Short-Chain Fatty Acid Production of Human Gut Microbiota

**DOI:** 10.3390/microorganisms10071268

**Published:** 2022-06-22

**Authors:** Sumudu Rajakaruna, Sergio Pérez-Burillo, Denise Lynette Kramer, José Ángel Rufián-Henares, Oleg Paliy

**Affiliations:** 1 Department of Biochemistry and Molecular Biology, Boonshoft School of Medicine, Wright State University, Dayton, OH 45435, USA; sumuduplus@gmail.com (S.R.); spburillo@gmail.com (S.P.-B.); denise.kramer@wright.edu (D.L.K.); 2 Departamento de Nutrición y Bromatología, Instituto de Nutrición y Tecnología de los Alimentos, Centro de Investigación Biomédica (CIBM), Universidad de Granada, 18071 Granada, Spain; jarufian@ugr.es

**Keywords:** melanoidins, gut microbiota, short-chain fatty acids, antioxidant capacity, human microbiome

## Abstract

Melanoidins are the products of the Maillard reaction between carbonyl and amino groups of macromolecules and are readily formed in foods, especially during heat treatment. In this study we utilized the three-stage Human Gut Simulator system to assess the effect of providing melanoidins extracted from either biscuits or bread crust to the human gut microbiota. Addition of melanoidins to the growth medium led to statistically significant alterations in the microbial community composition, and it increased short-chain fatty acid and antioxidant production by the microbiota. The magnitude of these changes was much higher for cultures grown with biscuit melanoidins. Several lines of evidence indicate that such differences between these melanoidin sources might be due to the presence of lipid components in biscuit melanoidin structures. Because melanoidins are largely not degraded by human gastrointestinal enzymes, they provide an additional source of microbiota-accessible nutrients to our gut microbes.

## 1. Introduction

Gut microbiota is known to affect host health at several different levels: by competing with pathogens, by interacting with the immune system, and by producing beneficial and detrimental metabolites [1]. One of the prominent roles of gut microbes is the digestion of certain food components. Gut microbes feed on the dietary components that remain undigested in the upper gut and reach the colon, particularly dietary fiber, and produce health-promoting short-chain fatty acids (SCFAs) [2].

Among the food components that are not digested by human gastrointestinal enzymes are melanoidins. These are high molecular weight organic compounds, which are the end products of the Maillard reaction that occurs between the carbonyl group of a reducing sugar and the amino group of an amino acid or a vitamin. This reaction can occur in foods during their heating and cooking, and is often manifested by a brown color (e.g., melanoidins are responsible for the color of toast). Melanoidins’ structure and composition are mostly undefined, largely due to the enormous variety of chemical structures that can be formed via the Maillard reaction among different food compounds [3,4]. For instance, coffee or cocoa melanoidins incorporate small phenolics, whereas bread and biscuit melanoidins are mainly formed by polysaccharides and proteins [3]. Fats can also play a relevant role in melanoidin formation since, once oxidized, their carbonyl group can easily react with an amino group of an amino acid [5].

Melanoidins are very common in processed foods with high sugar and/or protein content, such as biscuits and bread [6]. It is estimated that the daily melanoidin consumption by an average adult is around 10 g/day [3]. In the gastrointestinal tract, melanoidins resist digestion in the upper gut and reach the large intestine, where they are readily available for microbial fermentation [7]. In fact, melanoidins have been suggested as potential prebiotic compounds with health-promoting effects [8,9]. However, the use and transformation of melanoidins by gut microbiota is vastly unexplored, and only a handful of studies have been carried out so far. Bread melanoidins have been demonstrated to have prebiotic properties by stimulating the growth of beneficial *Bifidobacterium* bacteria [10]. In another study, in vitro batch fermentation of bread melanoidins using fecal material from healthy volunteers inhibited the growth of enteric bacteria [11]. Wu et al. [12] showed that diet supplementation of obese C57BL/6J mice with black garlic melanoidins led to a higher intestinal production of beneficial short-chain fatty acids and lower production of immunogenic lipopolysaccharide. Mice that were fed barley melanoidins had higher abundances of *Bifidobacterium* spp. and *Akkermansia* spp. [13].

In our previous study, using an in vitro batch fermentation, we investigated the utilization of commonly consumed melanoidins from different food sources by human fecal microbiota [14]. Based on their modulation of the microbiota community structure, different melanoidins could be distributed into several different groups. Digested melanoidins from bread crust, cereals, and pilsner and black beers promoted an expansion of the members of genus *Bifidobacterium*, whereas biscuit melanoidins promoted the expansion of *Faecalibacterium*. Fermentation of both melanoidin types led to an increased SCFA production in batch cultures [14].

Here, we expanded the previous study by assessing the effects of the addition of biscuit and bread crust melanoidins to human gut microbial communities maintained in the three-stage in vitro Human Gut Simulator (HGS), a validated model of the human colon [15]. We assessed gut microbial community structure and predicted functionality, production of short-chain fatty acids, and the antioxidant capacity of the cultures grown in a Western diet medium supplemented with either biscuit or bread crust melanoidins.

## 2. Materials and Methods

### 2.1. Melanoidin Extraction

Bread and biscuits were purchased locally from Granada, Spain. Bread crust and biscuit melanoprotein extractions were carried out using in vitro digestion with Pronase E as described [14]. Briefly, 250 g of either powdered bread crust or biscuits was mixed with 1.5 L of 400 U ml^−1^ Pronase E in 1.0 M phosphate buffer (pH 8.2). The mixture was stirred at 37 °C for 48 h. The insoluble fraction, containing polysaccharides and resistant starch, was removed by centrifugation. Fats were filtered out using Whatman filter paper. Melanoidins were concentrated from the remaining soluble fraction via diafiltration through a 5 kDa membrane, and then freeze-dried. The yield was between 7–8% for bread crust and 12–14% for biscuits. Several extractions were carried out for each melanoidin to obtain sufficient quantities.

### 2.2. In Vitro Human Gut Simulator Experiments

Gut microbial fermentation of melanoidins was carried out using the in vitro HGS system, which had been previously described [15]. To summarize, the HGS is an anaerobic system comprised of five sub-units: a medium reservoir, a proximal colon vessel, a transverse colon vessel, a distal colon vessel, and a waste collector. The medium reservoir supplies Western diet-influenced medium (WM) to the proximal colon vessel. WM has been designed to mimic the dietary contents that reach the colon of an adult after the digestion of a typical Western diet [15], and its composition is listed in Supplementary Table S1. All vessels were seeded with human microbiota inocula, which consisted of a thoroughly homogenized, equiweight mix of freshly collected fecal material from four healthy adults (two North Americans, one Western European, and one of South Asian ethnicity) with no recent use of antibiotics or dietary supplements, and no history of gastrointestinal diseases. The genus-level microbial composition of each fecal sample was assessed with 16S rRNA gene amplicon sequencing and is provided in Supplementary Table S2. The reservoir, vessels, and waste collector were connected by sterile tubing attached through peristaltic pumps, which ensured unidirectional, peristaltic movement of contents mimicking the movement of a food bolus in the gut. Each vessel was volume, temperature, and pH regulated to match the conditions of their respective colonic region, and the contents were agitated continuously. The system was sparged twice daily with a mixture of N_2_ and CO_2_ to ensure anaerobicity. The system was operated continuously for four weeks, and duplicate runs were performed for each type of melanoidin tested.

In each run, standard WM was provided for the first two weeks to establish a stable microbiota community in each region [15]. This was followed by the switch of the medium to WM plus melanoidins (7.0 g L^−1^, see Supplementary Table S1), and the system was maintained for an additional two weeks. Samples were drawn from each vessel regularly. Cell densities in each vessel were obtained via phase contrast microscopy by counting cells in four random quadrants of Spencer hemocytometer and averaging the results.

### 2.3. Microbial Genomic DNA Isolation and High Throughput Sequencing

Bacterial genomic DNA was isolated from samples using a ZR Fecal DNA MiniPrep kit (Zymo Research, Irvine, CA, USA). The 16S rRNA gene was amplified with two separate sets of degenerate primers targeting V1-V2 and V4 variable regions as we have done previously [14]. PCR amplification used 25 ng of starting genomic DNA, with ten linear cycles using only the forward primer, followed by 25 cycles of exponential PCR using both forward and reverse primers as described before [16]. The use of two different 16S rRNA gene regions and the incorporation of the linear PCR step reduced known high-throughput sequencing biases [17,18].

High throughput sequencing of the generated amplicons was carried out on an Ion Torrent Personal Genome Machine following the manufacturer’s protocols. After quality filtering, we obtained an average of 16,447 sequence reads for V1–V2 (range: 8668–37,788) and 25,167 reads for V4 (range: 13,667–74,026) regions. The full sequence dataset was deposited into the Sequence Read Archive repository (BioProject ID PRJNA824780). Obtained reads were processed in QIIME [19] using our default pipeline [20]. Taxon annotation was based on the Ribosomal Database Project Classifier v2.11 and RDP 16S rRNA training set 16. Obtained reads were converted into cell counts via the 16S rRNA gene copy adjustment procedure as we did before [15]. Independent cell counts obtained for both V1–V2 and V4 16S rRNA gene regions were merged using $C=\sqrt{(C_{V1V2}{}^{2}+C_{V4}{}^{2})/2}$ formula to calculate the final taxon abundance in each sample, where *C* is the cell count for each taxon. Cell counts of all samples were multiplied to match the cell density of each sample obtained via phase contrast microscopy as described above. This merged, cell density-adjusted dataset of cell counts was used for all downstream analyses.

### 2.4. Short-Chain Fatty Acids Measurements via HPLC-UV

HGS aliquots were centrifuged at 13,000× *g* for 2 min, and the supernatant was filtered through a 0.22 µm nylon filter. SCFAs were analyzed via HPLC-UV as described in Panzella et al. [21], using the Accela 600 HPLC system (Thermo Scientific, Waltham, MA, USA) equipped with a quaternary pump, an autosampler, and a UV-Vis photo diode array detector that was set to 210 nm. The analysis was carried out isocratically with a mobile phase composed of a mixture of 99% of solution A and 1% of solution B delivered at a flow rate of 0.250 mL min^−1^. Solution A was ultra-pure water acidified with 1% of formic acid, and solution B was acetonitrile acidified with 1% of formic acid. Compounds were separated on a 150 mm long reversed phase Accucore C18 column with particle size of 2.6 µm.

Six different SCFAs were analyzed: acetate, propionate, butyrate, lactate, succinate, and isovalerate. A calibration curve was generated for each acid using a concentration range from 0.002 mM to 100 mM. Results were expressed in mM.

### 2.5. Antioxidant Capacity Measurements via Ferric Reducing Ability of Plasma (FRAP)

The ability of sample supernatants to reduce Fe^3+^ to Fe^2+^ was measured via the FRAP method [22] adapted to a microplate reader. Briefly, 280 µL of freshly prepared FRAP solution and 20 µL of culture supernatant were added to a 96-well plate. FRAP solution was composed of 25 mL of 0.3 mM sodium acetate buffer (pH 3.6), 2.5 mL of a 10 mM solution of 2,4,6-tri(2-pyridyl)-s-triazine (TPTZ) dissolved in 40 mM HCl, and 2.5 mL of 20 mM FeCl_3_. Absorbance measurements were taken at 595 nm every 60 s for 30 min. A calibration curve was generated with Trolox standard with concentrations ranging from 0.01 to 0.4 mg mL^−1^. Results were calculated as mmol Trolox equivalents per liter.

### 2.6. Anaerobic Growth of Faecalibacterium, Bifidobacterium, and Roseburia on Melanoidins

*Bifidobacterium longum* subsp. *longum* DSM-20219 was obtained from the German Collection of Microorganisms and Cell Cultures (DSMZ). *Faecalibacterium prausnitzii* DSM-17677 and *Roseburia intestinalis* DSM-14610 were kindly provided by Dr. Eric Martens (University of Michigan, Ann Arbor, MI, USA). Strains were grown in Hungate tubes in 10 mL of Bifidobacterium medium (*B. longum*), YCFA (*F. prausnitzii*), or rumen bacteria medium (*R. intestinalis)* as recommended by DSMZ. Growth was carried out in an anaerobic chamber at 37 °C under the following gas mixture: 85% of N_2_, 10% of CO_2_, and 5% of H_2_. Once all cultures reached stationary phase (48 h), they were diluted 1:10 in anaerobic PBS, and 2 μL were subcultured into 198 μL of fresh medium in triplicate in a 96-well plate. Anaerobic growth of all cultures was then followed for 24 h in a microplate reader by measuring OD_600nm_.

### 2.7. Data Analyses

All statistical and multivariate analyses were carried out in R and Matlab [23]. We performed canonical correspondence analysis (CCA), using melanoidin supplementation and simulated colonic regions as explanatory variables, on both microbiota and SCFA profiles. Statistical significance and percent of explained variance were obtained with anova.cca function in R. UniFrac distance-based principal response curves (dbPRC) were used to interrogate temporal changes in microbiota composition upon melanoidin addition [24]. PICRUSt2 and STAMP software were used to analyze the predicted microbiota community functions [25]. Statistical significance of the predicted metagenome-based group separation in PCA space was based on the permutation of the Davies-Bouldin index as we described previously [26]. The distribution of PCA distances between group samples was evaluated using Welch’s *t*-test, using a previously employed approach [27]. Statistical significance of the differential pathway abundances between groups was calculated with Welch’s *t*-test with Benjamini-Hochberg correction for multiple hypothesis testing [28]. The statistical significance of the differences in measured values among vessels or media was calculated with repeated measures ANOVA.

## 3. Results

### 3.1. Growth of Diverse Human Gut Microbial Communities with and without Melanoidins

**Figure 1:**
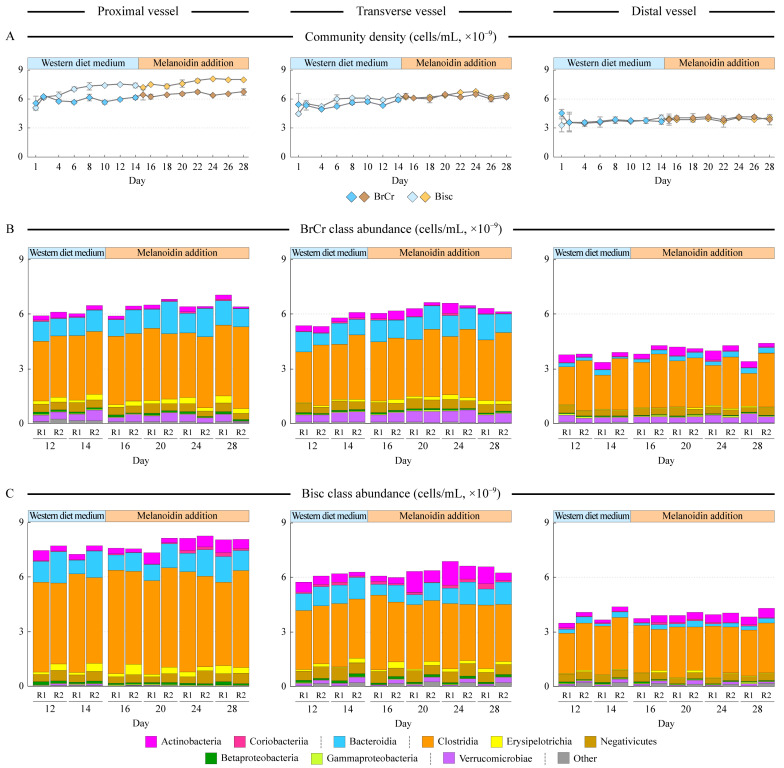
Dynamic changes in community density and composition. Different columns represent data for proximal, transverse, and distal colon simulating vessels as shown. The medium was switched from a standard Western diet medium (WM) to WM with additional melanoidins after taking samples on day 14. Panel (**A**) displays the cell density in each vessel. Error bars represent standard error of the mean (*n* = 2). Panels (**B**,**C**) show the cumulative abundance (absolute numbers of cells/mL of culture) of different bacterial classes at each time point in runs with either bread crust (BrCr, panel (**B**)) or biscuit (Bisc, panel (**C**)) melanoidins. R1 and R2 represent individual replicate runs.

Complex human gut microbial communities were maintained in the three-stage in vitro Human Gut Simulator over a four-week period. Communities in each vessel were established on the WM that resembled food bolus contents entering the colon on a typical Western diet [15]. As expected, the simulated proximal colon had the highest community density because the nutrients were provided directly to the first vessel only, followed by the transverse colon followed by the distal colon vessels (Figure 1A; *p* = 0.003, based on the repeated measures ANOVA). There were no statistically significant differences in the community richness and evenness among vessels at the genus abundance level. At the class level, all communities were dominated by Clostridia, followed by Bacteroidia (Figure 1B,C), matching our previous simulations in the HGS system [15,29].

Melanoidins, purified either from bread crust (BrCr) or from biscuits (Bisc), were added to the medium starting on day 15, and communities were followed for an additional two weeks. Addition of the melanoidins led to a minor, not statistically significant, increase in population density, and did not impact either community richness or evenness. Addition of melanoidins did not lead to remarkable changes in community composition at the class level, in part because melanoidins constituted just 18% of the total available macronutrients in the media. The most consistent change was the statistically significant increase in the abundance of Actinobacteria in all three HGS vessels upon the addition of biscuit melanoidins (1.8–2.0 fold increase; *p* = 0.015–0.049, Figure 1C). Addition of bread crust melanoidins led to a 22% increase of Verrucomicrobiae in the transverse vessel (*p* = 0.004) and a 3.4-fold increase in Erysipelotrichia in the distal vessel (*p* = 0.005).

### 3.2. Mucin Degradation Was Primarily Accomplished by Oribacterium and Olsenella Rather Than Akkermansia

We found a relatively low abundance of members of the class Verrucomicrobiae, and genus *Akkermansia* in particular, in all our runs. This was in contrast to our previous studies utilizing the HGS system [15,29]. Higher abundance of *Akkermansia*, a well-known mucin degrader, was expected, especially in the transverse and distal vessels, because we added mucins to each vessel twice a day to simulate their availability in the mucous layer of the colon. Thus, this peptidoglycan constitutes a substantial amount of overall nutrients available to microbiota in the transverse and distal vessels. It is likely that the mucin degradation role in our communities was filled by the members of genera *Oribacterium* and *Olsenella*. *Oribacterium* constituted 23.5%, 33.5%, and 40.5% of all cells (average over four weeks among four runs) in the proximal (PV), transverse (TV), and distal (DV) vessels, respectively. *Olsenella* accounted for 5.3%, 8.6%, and 9.1% cells, on average, in the three vessels. In contrast, *Akkermansia* comprised 2.7%, 5.4%, and 5.4% of all cells in the three vessels, respectively. Increase in the abundance of *Oribacterium* and *Olsenella* genera from the proximal to the transverse to the distal vessel was consistent with their ability to utilize mucins for growth. We found that genomes of the members of both genera encode various mucin degradation pathway enzymes as annotated in the UniProt [30] and String databases [31] (Supplementary Table S3). Several *Olsenella* species, isolated from other mammalian guts, were shown to be capable of degrading mucin [32]. We also found that genus *Oribacterium* was well represented in the microbiota community of the fecal inoculum donor of South Asian ethnicity (see Supplementary Table S2), which provided these bacteria to the initial seeding communities to replace *Akkermansia* as the predominant occupier of the mucin-degradation role. It appears that the mucin degradation niche might be fulfilled by different gut microbiota members in different human populations.

### 3.3. Conditions of Simulated Colon Regions and Addition of Melanoidins Significantly Affect Microbiota Structure and SCFA Production

**Figure 2:**
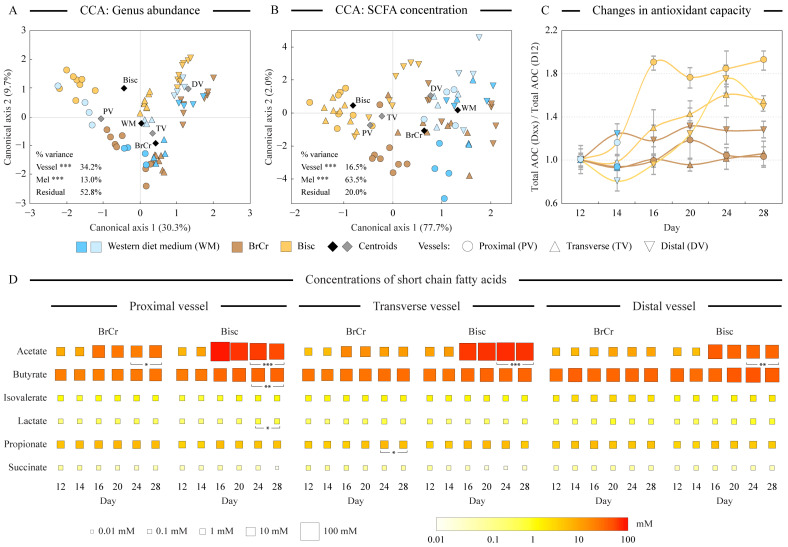
Analysis of community composition and metabolite production. Panels (**A**,**B**) display the output of the constrained canonical correspondence analysis (CCA) of the genus abundance (panel (**A**)) and short-chain fatty acid concentration (panel (**B**)) datasets. Medium type and vessel identity were used as explanatory variables that constrained the variability of each dataset. The percent of dataset variability explained by each axis is shown in parentheses in axis titles. For constraining categorical variables, the position of each class centroid is indicated with a diamond. The analysis of variance of the CCA outputs revealed the relative contribution of explanatory variables to the overall variability in the dataset; ***: *p* < 0.001. Panel (**C**) displays the change in the antioxidant capacity of cultures over time, defined as the ratio of total antioxidant capacity (AOC) in that sample divided by the total AOC from day 12 sample of the corresponding run. Error bars represent the standard error of the mean calculated for each ratio. Panel (**D**) visualizes the concentrations of measured short-chain fatty acids in all samples using color and size gradients as shown. Each square represents an average value between two replicate runs. Brackets denote cases where a statistically significant difference was calculated between the day 24 and day 28 values of a SCFA in comparison with day 12 and day 14 values. Significance levels: *: *p* < 0.050; **: *p* < 0.010; ***: *p* < 0.001.

We used constrained canonical correspondence analysis (CCA) of the genus abundance dataset to assess the relative effects of physicochemical differences among simulated colon regions and the addition of melanoidins to the medium on the overall variability in community composition profiles (Figure 2A). Differences in genus abundance among vessels contributed 34.2% to the overall dataset variance (*p* < 0.001), whereas the presence and identity of melanoidins in the medium explained 13.0% of the variance (*p* < 0.001). Concentrations of the most prevalent short-chain fatty acids known to be produced by human gut microbiota were determined by HPLC. Similar to the genus abundance-based CCA, CCA of SCFA values showed statistically significant effects (*p* < 0.001) of both the simulated colon region and the melanoidin addition on these volatile acid profiles (Figure 2B). Interestingly, in this case, the addition of melanoidins accounted for substantially higher variance in the SCFA dataset (63.5%) than it did in the differences among vessels (16.5%). One plausible explanation of this observation is that many different community members are able to ferment melanoidins in different vessels and replicate runs giving rise to lower consistency (and lower explained variance) of community composition changes.

Among measured SCFAs, acetate, butyrate, and propionate were the most abundant (Figure 2D). Total SCFA concentrations increased on average from the proximal to the transverse to the distal vessel on western diet medium (35.5 mM in PV, 39.8 mM in TV, and 45.4 mM in DV; *p* = 0.054), primarily due to a higher butyrate production in the latter two vessels. Addition of BrCr and Bisc to the medium had a different effect on the SCFA production (Figure 2D). Fermentation of biscuit melanoidins drastically increased the production of acetate, with concentrations increasing above six-fold in all three vessels (*p* < 0.005). Butyrate concentrations also increased 1.5-, 1.3-, and 1.3-fold in proximal, transverse, and distal vessels, respectively, compared to its production on WM (*p* = 0.008, 0.092, and 0.080, respectively). Propionate levels increased between 10 and 50% in different vessels, though the differences were not statistically significant. In contrast, the addition of BrCr to the medium had a substantially less profound effect on SCFA concentrations, with only acetate levels showing a statistically significant increase in the proximal colon vessel (3.1-fold increase, *p* = 0.011), and propionate increasing 1.5-fold in the transverse vessel (*p* = 0.050). Isovalerate, lactate, and succinate were present in relatively small amounts in all samples.

### 3.4. Changes in Antioxidant Capacity Differ between BrCr and Bisc Additions

Because melanoidins were shown previously to possess antioxidant properties [3], we measured total antioxidant capacity (AOC) in all vessels with and without melanoidin addition. While Bisc presence in the medium led to a statistically significant increase in total AOC in all three vessels (Figure 2C, 1.5–1.8-fold increase, *p* ≤ 0.036), BrCr melanoidins provided only a minor, not statistically significant, increase in AOC.

### 3.5. Temporal Changes in Microbiota Community Structure

**Figure 3:**
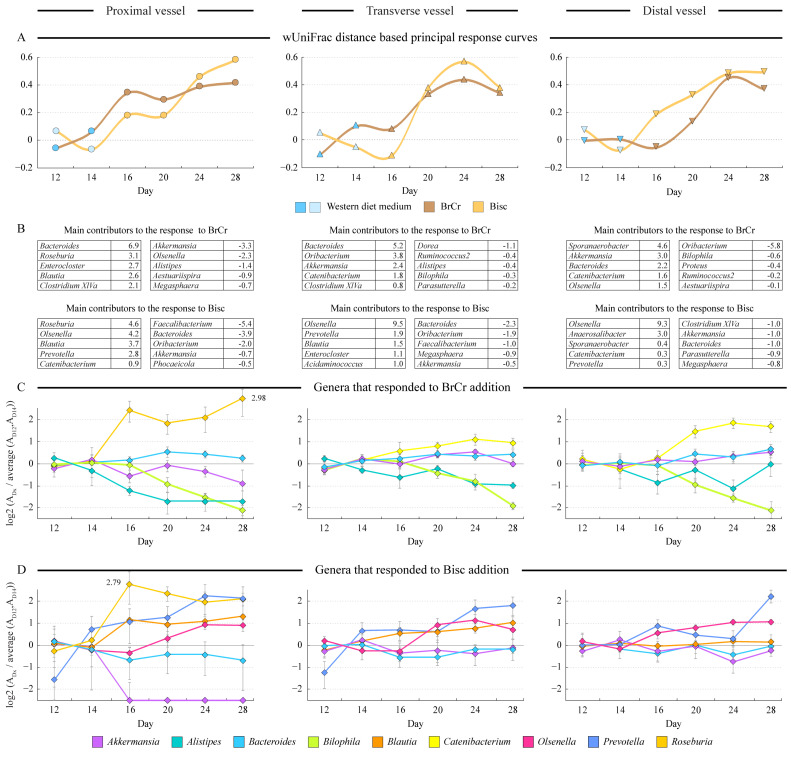
Longitudinal analysis of microbiota community changes upon melanoidin supplementation. Panel (**A**) displays the results of weighted UniFrac distance based principal response curves (dbPRC) analysis. Consensus community composition on days 12 and 14 (representing stabilized community on Western diet medium) were set as baseline for that run type (Bisc or BrCr) and were compared to the microbiota composition at every other time point. Larger deviation from zero on the Y axis represents greater shift of community structure from that of the baseline. The main microbial drivers of the observed shifts in the community composition are shown in the tables in panel (**B**). Positive numbers represent genera that increased in their abundance after medium switch; negative numbers represent genera that decreased. The change in abundance of several of these genera at different time points are shown in panels (**C**,**D**), defined as the log2 of the ratio of genus abundance in particular sample divided by the average of abundances on days 12 and 14. Value of one thus represents a two-fold increase in abundance. Error bars represent standard deviation of ratios calculated separately for each replicate run.

We used the weighted UniFrac distance-based principal response curves algorithm (dbPRC) [24] to assess temporal divergence of community structure in each vessel upon melanoidin addition. Both melanoidins elicited gradually increasing changes in the microbiota composition in all three vessels (Figure 3A), with Bisc prompting somewhat larger divergence. As expected, community composition changes upon melanoidin addition were observed first in the PV followed by TV and eventually by DV communities. The dbPRC algorithm also determined the relative contributions of different genera to the community composition changes from the baseline. The top ten contributing genera (five decreasing and five increasing in abundance) in each vessel are listed in Figure 3B, and their abundances in samples are provided in Supplementary Table S4. Addition of Bisc consistently increased the cell counts for *Prevotella*, *Olsenella*, and *Blautia* in all three vessels (see Figure 3D). *Bacteroides*, *Akkermansia*, *Oribacterium*, *Faecalibacterium*, and *Megasphera* were the top decreasing genera. There was less consistency among different communities in their response to BrCr melanoidins. *Bacteroides*, *Catenibacterium*, and *Blautia* showed the most consistent increases, whereas *Bilophila* and *Alistipes* showed the most consistent decreases (Figure 3C). Both Bisc and BrCr caused significant increases in *Roseburia* abundance specifically in the proximal vessel. *Akkermansia* had very inconsistent responses between different melanoidins and among different vessels, possibly due to the confounding effect of its competition for mucin components with *Oribacterium* and *Olsenella* as we described above.

### 3.6. Changes in Predicted Community-Encoded Functions upon Melanoidin Addition

**Figure 4:**
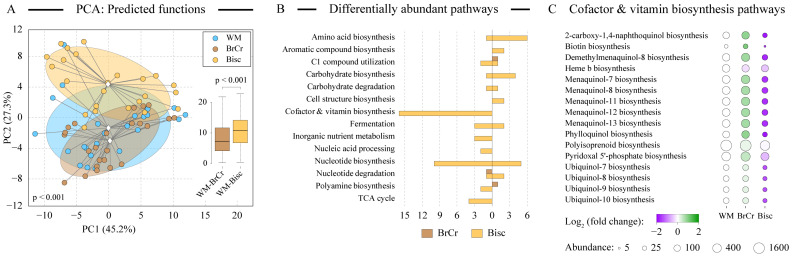
Analysis of predicted functional capacities of microbial communities. Panel (**A**) depicts the results of the principal components analysis (PCA) of predicted metagenome-encoded microbial functions. The percent of dataset variability explained by each axis is shown in parentheses in axis titles. Individual samples are colored as shown and are linked to group centroid. Group clouds show areas of three standard errors around the group centroid (diamond). The distributions of pair-wise distances in PCA space between positions of samples from different groups are shown as boxplots. Statistical significance was calculated by Welch’s *t*-test. Panel (**B**) visualizes the numbers of statistically differentially abundant (DA) pathways in the predicted metagenomes of Bisc and BrCr grown communities compared to the WM communities. Predicted functional genes were assigned to pathways and pathway groups based on MetaCyc database [33]. Only pathway groups with at least two DA pathways are shown. There were also 14 pathway groups with a single DA pathway (13 for Bisc comparison and 1 for BrCr comparison). Pathways that increased in predicted genome encoding in Bisc or BrCr samples in comparison to WM samples are shown to the right, and pathways that decreased—to the left side from zero. The prevalence of individual pathways within cofactor and vitamin biosynthesis pathway groups is displayed in panel (**C**). The size of each circle is proportionate to the geometric mean of PICRUSt2-predicted pathway gene abundances among metagenomes in that sample category. Circle color represents the magnitude of the ratio of pathway abundance between BrCr or Bisc samples and the baseline WM community.

We used the PICRUSt2 algorithm to evaluate the predicted functions of profiled microbial communities. Overall, predicted metagenomes of WM and BrCr samples were positioned together in the PCA space (Figure 4A), whereas there was a statistically significant separation of Bisc and WM samples (Figure 4A, *p* < 0.001). The distribution of WM-Bisc and WM-BrCr distances in the PCA space was also distinct with statistical significance (see insert in Figure 4A; *p* < 0.001 based on Welch’s *t*-test). Consistent with this finding, a substantial number of specific pathways were differentially encoded in the predicted metagenomes of Bisc-grown communities compared with WM-grown cultures (Figure 4B). The largest category was cofactor and vitamin biosynthesis, with 16 different pathways all being less represented in the communities maintained in the presence of biscuit melanoidins. The majority of these coded for the biosynthesis of lipid-soluble coenzyme Q and vitamin K compounds (between 2.8 and 3.9-fold decrease in Bisc; Figure 4C).

Genes of the pyridoxal 5’-phosphate biosynthesis were also less abundant in Bisc metagenomes. Pyridoxamine (one of vitamin B6 forms) can form adducts with Amadori products such as melanoidins, and vitamin B6 is present in small amounts in “Marie” biscuits that we used to isolate Bisc melanoidins from. Other pathway categories differentially abundant in Bisc samples compared with Western diet medium cultures included amino acid and carbohydrate biosynthesis (most increased in prevalence) and nucleotide biosynthesis (abundance of ten pathways decreased and five increased; see Figure 4B). In contrast, only a few pathways showed any differential abundance in BrCr samples compared with WM, consistent with our other findings revealing a generally muted response of human gut microbiota to bread crust melanoidin addition.

## 4. Discussion

In the current study we assessed the alterations of the human gut microbiota upon medium supplementation with two types of melanoidins—those isolated from biscuits and from bread crust. Overall, both microbiota structure and their SCFA production changed significantly upon melanoidin supplementation (see Figure 2A,B). However, the magnitude of these alterations contrasted between the studied melanoidins. Biscuit melanoidins elicited significant changes in many of the studied parameters including microbiota structure, its predicted functional capacity, and the production of short-chain fatty acids and antioxidants, with many of these changes known to be associated with health benefits to the host. Specifically, upon Bisc addition to the growth medium, the abundance of class Actinobacteria increased 1.8–2.0-fold in all three simulated colon regions (see Figure 1C). Acetate production increased above 6-fold in all vessels and that of butyrate 1.5-fold in the proximal vessel (Figure 2D). Total antioxidant capacity also increased 1.5–1.9-fold in all three vessels supplied with Bisc melanoidins (Figure 2C).

In contrast, the presence of bread crust melanoidins resulted in more subdued responses, either revealing lower magnitude of the differences or not achieving statistical significance of the changes. BrCr were still utilized by human gut microbiota, as evidenced by the slight increase in community density, and elevation in the total production of SCFAs, especially in the proximal vessel (Figure 2D).

What might explain these observed disparities in Bisc and BrCr fermentation? One notable difference between bread crust and biscuit products was the substantial presence of dietary fats in the latter. Fats constituted 15% of biscuits by weight, mostly due to the use of high oleic acid sunflower oil (as listed on the product dietary label). Purchased bread, on the other hand, only contained 1.5% by weight as dietary fats. We revealed several lines of evidence, which suggested that some of these fatty acids were incorporated into melanoidin structure during biscuit preparation and were subsequently used by our microbial communities. First, it has been shown previously that lipids can participate in the melanoidin-forming condensation reactions via their carbonyl groups [34]. Second, acetate, which is the primary end product of fatty acyl beta oxidation, accounted for the majority of the SCFA production increase on Bisc (Figure 2D). Third, the reduced encoding of lipid-soluble vitamins by microbiota grown on Bisc might be explained by the incorporation of these compounds in the hydrophobic regions of fatty acyl tails of the complex melanoidin structures. Therefore, these vitamins can be co-extracted with melanoidins during purification, and become available to the microbiota community, reducing the need to encode biosynthesis of these vitamins in the bacterial genomes. Fourth, substantial alterations to the predicted abundance of central metabolism pathways were also observed in Bisc-maintained cultures, including carbohydrate, amino acid, and nucleotide biosynthesis pathways, consistent with the increased presence of lipids in the environment.

This hypothesized presence of lipid moieties in the Bisc melanoidin structures did not lead, however, to the increased abundance of the lipidophilic microbes such as *Bilophila* and *Alistipes*, which we identified previously as being able to grow on dietary fatty acids [15]. Instead, several other genera increased their cell counts in the presence of biscuit melanoidins including *Prevotella*, *Blautia*, and *Olsenella* (see Figure 3D). *Roseburia*, a prominent polysaccharide degrader, increased in the proximal vessel in the presence of either BrCr or Bisc melanoidins, and bread crust melanoidins promoted the expansion of *Catenibacterium* in the transverse and distal vessels (see Figure 3C).

In our previous analysis of human gut microbiota fermentation of food melanoidins performed in batch cultures, we identified members of genera *Bifidobacterium* and *Faecalibacterium* as dominant members of communities on BrCr and Bisc melanoidins, respectively [14]. Surprisingly, these genera were not abundant on the corresponding media in our HGS communities; indeed, they were present at low numbers in all runs. To test whether such discrepancy might be associated with specific differences in environmental conditions and initial community composition between studies, we grew monocultures of *Bifidobacterium longum* and *Faecalibacterium prausnitzii* anaerobically in their recommended growth media and in variants where all carbon sources were replaced with either biscuit or bread crust melanoidins. As shown in Table 1, *F. prausnitzii* grew in the Bisc-containing medium but did not grow in the BrCr-based medium. *B. longum* achieved higher stationary phase cell density in BrCr cultures. These data are consistent with our previous findings [14]. To provide further support for the findings from our HGS experiments, *Roseburia intestinalis*, which was found to increase on both melanoidins in our HGS experiments, grew well on each melanoidin as the only readily available source of carbon (Table 1).

There are several plausible explanations of the observed differences in *Bifidobacterium* and *Faecalibacterium* abundance between HGS and batch fermentation results. First, the experimental platforms are very different, and multi-stage gut simulating systems have been shown to establish microbiota communities distinct from those that used a single fermentation chamber or tube [35]. Second, the pH was not controlled in the previous batch fermentation experiments, and our tests showed that by 24 h of incubation the medium reached approximately pH 4.5 in such batch system. In contrast, the pH of each HGS vessel was maintained at pH 6.0, 6.5, and 7.0 in the proximal, transverse, and distal vessels, respectively, with a range of ± 0.1. Third, all media used a combination of five abundant dietary fatty acids as sources of fats. Several of these fatty acids were recently shown to be inhibitory to *Bifidobacterium* growth [36], likely explaining the low abundance of this genus in our runs. In addition, both *Bifidobacterium* and *Faecalibacterium* are adversely affected by bile salts [36,37] that were included in our media to solubilize lipids. Fourth, the sources (human volunteers) used to generate fecal inocula to seed HGS and fermentation tubes were different, and likely led to a different starting community in these experiments. The latter hypothesis is supported by our finding that in our HGS experiments mucin degradation niche was occupied by members of *Oribacterium* and *Olsenella*, because they were highly abundant in the fecal microbiota of one of the fecal donors.

Most foods within the Western diet are heat-processed, so that a large amount of melanoidins is produced and consumed as part of the human diet [38]. Although melanoidins have been previously described as health-promoting compounds due to their chemopreventive and antioxidant capacity, here we show that bread and biscuits melanoidins modify both the composition and functionality of the human gut microbiota. The results from the present study support the idea that melanoidins, which mostly escape human digestion, promote the generation of large amounts of short-chain fatty acids by human gut microbiota, which in turn will benefit host health. Unraveling such beneficial effects of melanoidins does not imply, however, that foods should be burned, as along the development of the Maillard reaction, many toxic compounds (e.g., acrylamide, heterocyclic amines) are also produced during food heating. Our findings call for further technological efforts to study and affect the formation of these compounds during food processing, especially in cereal-derived foods.

## Figures and Tables

**Table 1:** Steady state culture densities of select bacterial species grown anaerobically in batch culture.

Organism	Standard Medium *^,^^†^	Bisc *	BrCr *	YEM *^,^^‡^
*Bifidobacterium longum*	0.55 ± 0.02	0.22 ± 0.02	0.31 ± 0.02	ng **
*Faecalibacterium prausnitzii*	0.49 ± 0.01	0.15 ± 0.02	ng	ng
*Roseburia intestinalis*	0.19 ± 0.06	0.15 ± 0.02	0.15 ± 0.02	ng

* Data represent OD_600nm_ measurements of culture density and are shown as arithmetic mean (*n* = 3) ± standard deviation. ^†^ Standard growth media used were Bifidobacterium medium (*B. longum*), YCFA (*F. prausnitzii*), and rumen bacteria medium (*R. intestinalis*). ^‡^ YEM—standard growth medium with no carbon sources outside of yeast extract. ** ng—no growth.

## Data Availability

The full 16S rRNA amplicon sequence dataset was deposited into the Sequence Read Archive repository (BioProject ID PRJNA824780).

## Supplementary Materials

The following supporting information can be downloaded at: https://www.mdpi.com/article/10.3390/microorganisms10071268/s1, Table S1: chemical composition of the media used to maintain HGS cultures, Table S2: relative abundances of microbial genera in the fecal microbiota of inoculum donors, Table S3: cell densities of genera listed as main contributors to the observed community changes in Figure 3B, Table S4: analysis of mucin degradation pathway genes encoded in the genomes of select *Akkermansia*, *Olsenella*, and *Oribacterium* species. Reference [39] is cited in Supplementary Materials.
