# Supplementary material for: Dietary Melanoidins from Biscuits and Bread Crust Alter the Structure and Short-Chain Fatty Acid Production of Human Gut Microbiota
Source: Microorganisms. 2022 Jun 22;10(7):1268. doi: 10.3390/microorganisms10071268 (PMC9323165; doi:10.3390/microorganisms10071268)
Supplement: Supplementary file 1 [file microorganisms-10-01268-s001.zip › microorganisms-1710655-supplementary.pdf]

Supplementary Table S1. Medium composition, g l^{-1}

Medium component	*WM**	*MEL**
Carbohydrates		
Arabinogalactan	1.8	1.8
Guar gum	0.9	0.9
Inulin	0.9	0.9
Pectin	1.8	1.8
Starch	4.4	4.4
Xylan	0.9	0.9
Cellobiose	0.9	0.9
Glucose	0.5	0.5
Fructose	0.5	0.5
Proteins		
Peptone	3.3	3.3
Casein	2.0	2.0
Lipids		
Capric acid (C10:0)	0.3	0.3
Palmitic acid (C16:0)	1.5	1.5
Stearic acid (C18:0)	0.7	0.7
Oleic acid (C18:1)	1.8	1.8
Linoleic acid (C18:2)	1.1	1.1
Mucin (porcine gastric)	*4.0*	*4.0*
Melanoidins (BrCr or Bisc)	-	*7.0*
Yeast extract	*3.0*	*3.0*
Vitamins	*1.0*	*1.0*
Salts, other components	*14.9*	*14.9*
Bile salts	1.0	1.0

* WM – Western diet medium, MEL – WM with additional melanoidins added as shown

Supplementary Table S2

Taxon	Donor 1	Donor 2	Donor 3	Donor 4
Acetivibrio	0.08%	0.06%	0.00%	0.13%
Acetobacteroides	0.00%	0.18%	0.00%	0.00%
Acidaminococcus	0.19%	0.00%	0.28%	0.00%
Acutalibacter	0.16%	0.00%	0.00%	0.02%
Adlercreutzia	0.00%	0.00%	0.00%	0.02%
Aestuariispira	0.00%	0.01%	0.00%	0.00%
Agathobacter	0.37%	1.67%	0.62%	0.74%
Agathobaculum	0.00%	0.00%	0.00%	0.02%
Akkermansia	0.00%	0.54%	0.00%	0.40%
Alistipes	0.57%	0.80%	0.07%	0.78%
Alloprevotella	0.00%	0.00%	2.36%	0.00%
Anaerobacter	0.03%	0.00%	0.00%	0.03%
Anaerobacterium	0.00%	1.39%	0.00%	0.00%
Anaerobutyricum	0.57%	0.72%	0.40%	0.44%
Anaeromassilibacillus	0.00%	0.00%	0.03%	0.00%
Anaeroplasma	0.00%	0.33%	0.00%	0.00%
Anaerostipes	3.32%	0.61%	1.75%	1.58%
Anaerotignum	0.00%	0.00%	0.00%	0.02%
Anaerotruncus	0.00%	0.16%	0.00%	0.00%
Anaerovorax	0.00%	0.11%	0.00%	0.12%
Bacteroides	6.59%	3.00%	1.91%	5.05%
Bariatricus	0.03%	0.10%	0.00%	0.05%
Barnesiella	0.08%	0.11%	0.00%	0.20%
Bifidobacterium	1.26%	0.54%	5.14%	1.82%
Bilophila	0.08%	0.01%	0.00%	0.03%
Blautia	10.27%	3.93%	5.38%	9.75%
Bulleidia	0.00%	0.01%	0.00%	0.00%
Butyricicoccus	0.25%	0.00%	0.09%	0.18%
Butyricimonas	0.00%	0.18%	0.03%	0.06%
Butyrivibrio	0.00%	0.06%	0.03%	0.04%
Catenibacterium	0.00%	0.00%	6.68%	0.00%
Christensenella	0.08%	0.08%	0.00%	0.07%
Citrobacter	0.00%	0.00%	0.05%	0.00%
Cloacibacillus	0.00%	0.01%	0.00%	0.00%
ClostridiumIV	0.28%	1.79%	0.00%	1.91%
Clostridium sensu stricto	0.08%	0.06%	0.00%	0.11%
ClostridiumXI	0.08%	0.00%	0.00%	0.03%
ClostridiumXlVa	0.76%	0.53%	0.14%	0.30%
ClostridiumXlVb	0.24%	0.04%	0.03%	0.03%
ClostridiumXVIII	0.31%	0.11%	0.00%	0.34%
Collinsella	0.16%	0.39%	2.90%	2.48%
Coprobacillus	0.00%	0.00%	0.00%	0.03%
Coprobacter	0.00%	0.03%	0.00%	0.03%
Coprococcus	1.02%	2.44%	0.83%	2.55%
Cuneatibacter	0.00%	0.00%	0.00%	0.03%
Desulfovibrio	0.00%	0.05%	0.00%	0.00%
Dialister	0.08%	0.00%	1.22%	0.10%
Dorea	0.83%	1.45%	2.20%	1.83%
Eggerthella	0.09%	0.00%	0.00%	0.00%
Eisenbergiella	0.00%	0.07%	0.00%	0.00%
Enterobacter	0.00%	0.00%	0.05%	0.00%
Enterococcus	0.08%	0.05%	0.05%	0.00%
Erysipelotrichaceae incertae sedis	0.00%	0.05%	0.00%	0.06%
Escherichia/Shigella	0.00%	0.04%	0.19%	0.06%
Ethanoligenens	0.00%	0.00%	0.00%	0.03%
Faecalibacillus	0.09%	0.14%	0.00%	0.05%
Faecalibacterium	35.79%	8.64%	4.83%	34.69%
Faecalicoccus	0.00%	0.01%	0.00%	0.00%

Taxon	Donor 1	Donor 2	Donor 3	Donor 4
Faecalitalea	0.08%	0.20%	0.05%	0.00%
Flavonifractor	0.09%	0.11%	0.00%	0.07%
Fusicatenibacter	7.08%	2.25%	0.72%	3.21%
Gemmiger	0.27%	1.45%	0.46%	0.45%
Granulicatella	0.08%	0.00%	0.00%	0.00%
Haemophilus	0.00%	0.00%	0.03%	0.00%
Holdemanella	0.00%	3.20%	4.79%	0.00%
Howardella	0.09%	0.06%	0.00%	0.02%
Hungatella	0.00%	0.00%	0.00%	0.02%
Intestinibacter	0.08%	0.05%	0.13%	0.05%
Intestinimonas	0.00%	0.00%	0.03%	0.00%
Klebsiella	0.08%	0.00%	0.00%	0.00%
Lachnospira	0.00%	0.03%	0.00%	0.08%
Lachnospiracea incertae sedis	2.72%	3.22%	1.36%	3.56%
Lactobacillus	0.00%	0.00%	4.70%	0.02%
Ligilactobacillus	0.00%	0.00%	0.46%	0.00%
Lysinibacillus	0.00%	0.00%	0.03%	0.00%
Marseilla	0.00%	0.05%	0.00%	0.00%
Massiliprevotella	0.00%	0.00%	0.14%	0.00%
Mediterraneibacter	0.25%	0.17%	0.05%	0.09%
Megasphaera	0.00%	0.00%	0.44%	0.00%
Merdimonas	0.00%	0.00%	0.00%	0.05%
Methanobrevibacter	0.00%	0.78%	0.00%	0.11%
Mitsuokella	0.00%	0.82%	0.00%	0.03%
Mobilitalea	0.00%	0.11%	0.00%	0.00%
Monoglobus	0.00%	0.00%	0.00%	0.02%
Murimonas	0.00%	0.03%	0.00%	0.00%
Neglecta	0.03%	0.07%	0.00%	0.03%
Odoribacter	0.01%	0.06%	0.00%	0.06%
Oribacterium	0.00%	0.00%	4.78%	0.00%
Oscillibacter	0.16%	0.80%	0.05%	0.96%
Papillibacter	0.00%	0.01%	0.00%	0.03%
Parabacteroides	1.43%	0.69%	0.30%	0.24%
Paraprevotella	0.00%	0.59%	0.00%	0.02%
Parasutterella	0.00%	0.05%	0.44%	0.22%
Peptococcus	0.00%	0.00%	0.10%	0.00%
Phascolarctobacterium	0.15%	0.13%	0.00%	0.00%
Phocaeicola	0.43%	0.49%	0.14%	0.25%
Prevotella	0.08%	1.58%	27.27%	0.05%
Pseudocitrobacter	0.00%	0.00%	0.05%	0.00%
Pseudoflavonifractor	0.00%	0.05%	0.00%	0.03%
Raoultibacter	0.00%	0.00%	0.00%	0.02%
Robinsoniella	0.00%	0.05%	0.00%	0.02%
Romboutsia	0.98%	0.28%	0.03%	0.62%
Roseburia	6.34%	8.60%	6.62%	3.04%
Ruminococcus	0.91%	5.06%	0.09%	3.41%
Ruminococcus2	2.47%	1.13%	1.51%	1.34%
Saccharofermentans	0.00%	0.05%	0.00%	0.05%
Senegalimassilia	0.00%	0.00%	0.09%	0.00%
Slackia	0.00%	0.03%	0.00%	0.02%
Sporobacter	0.00%	0.10%	0.00%	0.03%
Stenotrophomonas	0.00%	0.00%	0.00%	0.02%
Streptococcus	0.18%	0.20%	0.25%	0.03%
Subdoligranulum	0.09%	0.08%	0.03%	0.14%
Sutterella	0.00%	0.11%	0.00%	0.00%
Terrisporobacter	0.08%	0.00%	0.00%	0.03%
Turicibacter	0.08%	0.06%	0.00%	0.00%
Unassigned	11.91%	36.63%	7.63%	15.35%
Vampirovibrio	0.03%	0.06%	0.00%	0.02%

Data are shown as relative abundance of each genus within its community

Supplementary Table S3

**Enzymes of mucin degradation pathways ***	**Presence in**		
	A. muciniphila	***Olsenella* taxa**	***Oribacterium* taxa**
α-galactosidase	Akk	Ols 1,Ols 3, Ols2, Ols 5	Ori1, Ori 2, Ori 3
α-L-arabinofuranosidase			Ori 2
α-L-fucosidase	Akk	Ols 1, Ols 4	Ori 2, Ori 3
α-mannosidase			Ori 1
β-galactosidase	Akk	Ols 1, Ols 3	Ori 2, Ori 3
Sialidase		Ols 1	
Sulfatase	Akk	Ols 1,Ols 3, Ols2, Ols 5	Ori 1, Ori 3

**Taxon name**	**UniProt Taxon ID**	**Designation**
A. muciniphila ATCC BAA-835	349741	Akk
Olsenella sp. An290	1965625	Ols 1
Olsenella sp. An270	1965615	Ols 2
Olsenella sp. An293	1965626	Ols 3
Olsenella sp. DNF00959	1476999	Ols 4
Olsenella sp. oral taxon 807	712411	Ols 5
Oribacterium sinus F0268	585501	Ori 1
Oribacterium sp.	1969407	Ori 2
Oribacterium sp. C9	1943579	Ori 3

*** Reference:**

Tailford LE, Crost EH, Kavanaugh D, Juge N. Mucin glycan foraging in the human gut microbiome. Front Genet. 2015 Mar 19;6:81. doi: 10.3389/fgene.2015.00081

Supplementary Table S4

		Main contributors to the response in Proximal Vessel communities (cells/ml, $\times 10^9$)										
	**Increased**	*Bacteroides*	*Roseburia*	*Enterocloster*	*Blautia*	*Clostridium XlVa*	**Decreased**	*Akkermansia*	*Olsenella*	*Alistipes*	*Aestuariispira*	*Megasphaera*
**BrCr**	Day 12	0.83	0.04	0.16	0.19	0.19	Day 12	0.71	0.68	0.17	0.15	0.21
	Day 14	0.91	0.05	0.20	0.20	0.18	Day 14	0.94	0.48	0.11	0.11	0.22
	Day 16	0.98	0.26	0.32	0.46	0.24	Day 16	0.56	0.47	0.06	0.07	0.16
	Day 20	1.27	0.17	0.42	0.36	0.41	Day 20	0.78	0.37	0.04	0.09	0.19
	Day 24	1.18	0.20	0.44	0.30	0.41	Day 24	0.64	0.43	0.04	0.07	0.14
	Day 28	1.03	0.37	0.29	0.38	0.26	Day 28	0.44	0.43	0.04	0.05	0.18
		Roseburia	*Olsenella*	*Blautia*	*Prevotella*	*Catenibacterium*		*Faecalibacterium*	*Bacteroides*	*Oribacterium*	*Akkermansia*	*Phocaeicola*
**Bisc**	Day 12	0.07	0.47	0.20	0.02	0.24	Day 12	1.70	1.23	1.62	0.05	0.13
	Day 14	0.10	0.35	0.18	0.10	0.27	Day 14	2.17	0.87	1.52	0.05	0.10
	Day 16	0.57	0.32	0.43	0.13	0.37	Day 16	1.64	0.68	1.58	0.00	0.08
	Day 20	0.42	0.51	0.37	0.15	0.24	Day 20	2.01	0.81	1.26	0.00	0.09
	Day 24	0.32	0.79	0.41	0.29	0.25	Day 24	1.68	0.83	1.48	0.00	0.07
	Day 28	0.36	0.77	0.48	0.27	0.36	Day 28	1.35	0.80	1.42	0.00	0.09

		Main contributors to the response in Transverse Vessel communities (cells/ml, $\times 10^9$)										
	**Increased**	*Bacteroides*	*Oribacterium*	*Akkermansia*	*Catenibacterium*	*Clostridium XlVa*	**Decreased**	*Dorea*	*Ruminococcus2*	*Alistipes*	*Bilophila*	*Parasutterella*
**BrCr**	Day 12	1.44	4.00	0.75	0.16	0.11	Day 12	0.36	0.09	0.11	0.05	0.13
	Day 14	1.74	4.33	1.05	0.21	0.15	Day 14	0.28	0.06	0.08	0.07	0.14
	Day 16	1.88	4.39	0.89	0.28	0.16	Day 16	0.20	0.05	0.06	0.06	0.12
	Day 20	2.16	4.09	1.19	0.33	0.24	Day 20	0.29	0.05	0.08	0.05	0.13
	Day 24	2.03	4.60	1.31	0.40	0.21	Day 24	0.21	0.04	0.05	0.04	0.13
	Day 28	2.14	4.86	0.90	0.36	0.18	Day 28	0.18	0.02	0.05	0.02	0.10
		Olsenella	*Prevotella*	*Blautia*	*Enterocloster*	*Acidaminococcus*		*Bacteroides*	*Oribacterium*	*Faecalibacterium*	*Megasphaera*	*Akkermansia*
**Bisc**	Day 12	1.20	0.05	0.19	0.18	0.58	Day 12	1.61	3.73	0.29	0.31	0.30
	Day 14	0.87	0.19	0.24	0.13	0.67	Day 14	1.67	3.96	0.35	0.38	0.42
	Day 16	0.86	0.19	0.31	0.13	0.65	Day 16	1.12	4.47	0.37	0.27	0.28
	Day 20	1.96	0.18	0.34	0.27	0.72	Day 20	1.13	3.78	0.29	0.23	0.30
	Day 24	2.28	0.38	0.36	0.29	0.81	Day 24	1.44	3.66	0.21	0.26	0.28
	Day 28	1.71	0.38	0.44	0.26	0.64	Day 28	1.45	3.63	0.16	0.23	0.33

		Main contributors to the response in Distal Vessel communities (cells/ml, $\times 10^9$)										
	**Increased**	*Sporanaerobacter*	*Akkermansia*	*Bacteroides*	*Catenibacterium*	*Olsenella*	**Decreased**	*Oribacterium*	*Bilophila*	*Proteus*	*Ruminococcus2*	*Aestuariispira*
**BrCr**	Day 12	0.27	0.60	0.30	0.05	0.65	Day 12	3.30	0.05	0.05	0.05	0.02
	Day 14	0.36	0.52	0.33	0.03	0.64	Day 14	3.14	0.05	0.04	0.02	0.02
	Day 16	0.49	0.64	0.30	0.05	0.52	Day 16	3.68	0.05	0.04	0.05	0.01
	Day 20	0.45	0.60	0.43	0.11	0.66	Day 20	3.28	0.03	0.03	0.03	0.02
	Day 24	0.64	0.72	0.39	0.14	0.88	Day 24	2.87	0.02	0.02	0.03	0.01
	Day 28	0.57	0.80	0.50	0.13	0.55	Day 28	2.84	0.01	0.02	0.02	0.00
		Olsenella	*Anaerosalibacter*	*Sporanaerobacter*	*Catenibacterium*	*Prevotella*		*Clostridium XlVa*	*Akkermansia*	*Bacteroides*	*Parasutterella*	*Megasphaera*
**Bisc**	Day 12	0.56	0.12	0.18	0.09	0.01	Day 12	0.19	0.19	0.41	0.10	0.18
	Day 14	0.44	0.14	0.17	0.06	0.01	Day 14	0.20	0.27	0.32	0.10	0.17
	Day 16	0.73	0.16	0.13	0.16	0.02	Day 16	0.15	0.19	0.28	0.09	0.20
	Day 20	0.87	0.21	0.13	0.12	0.01	Day 20	0.12	0.22	0.36	0.06	0.13
	Day 24	1.03	0.29	0.24	0.07	0.01	Day 24	0.15	0.14	0.27	0.04	0.13
	Day 28	1.04	0.33	0.23	0.06	0.04	Day 28	0.15	0.19	0.35	0.05	0.12
